# Factors associated with home delivery in Bahirdar, Ethiopia: A case control study

**DOI:** 10.1186/1756-0500-5-653

**Published:** 2012-11-24

**Authors:** Fantu Abebe, Yemane Berhane, Belaineh Girma

**Affiliations:** 1 Amhara National Regional Health Bureau, Bahir Dar, Ethiopia; 2 Addis Continental Institute of Public Health, Addis Ababa, Ethiopia

## Abstract

**Background:**

In Ethiopia although pregnant mothers increasingly attend antenatal clinics, utilization of skilled delivery service remains very low. The individual or health system factors that affect women’s preferences for delivery places are not well known.

**Method:**

A case control study was conducted in July 2010 to assess factors associated with utilization of institutional delivery service. A total of 324 mothers who recently delivered and visited either postnatal care or sought immunization services were included. Cases (n = 108) were mothers who gave birth at home and controls (n = 216) were those who delivered at health facility. Pre-tested and standardized questionnaires were used to collect relevant data by trained data collectors. Logistic regression model was used to control for confounding.

**Result:**

The likelihood of delivering at home was greater among mothers with inadequate knowledge of pregnancy related services (AOR = 62, 95% CI: 3, 128.4), those who started attending ANC after 24 weeks of gestation (AOR 8.7, 95% CI: 2.2, 33.3), mothers having no formal education (Adjusted OR 4.2, 95% CI 1.63, 11.27) and rural residents (AOR = 3.6, 95%CI: 1.4, 9.0).

**Conclusion:**

The predominant factors associated with home delivery services were lack of knowledge about obstetrics care, delay in starting Antenatal Care (ANC) follow up, having, Illiteracy and rural residence. Audience specific behavioral change communication should be designed to improve the demand for delivery services. Health professionals should take the opportunity to encourage mothers attend delivery services during ANC follow up. Improvements should be made in social conditions including literacy and major social mobilization endeavors.

## Background

In Ethiopia, the majority of births occur without the help of a skilled assistant (defined as a midwife, nurse trained, or a doctor) and mainly at home [1]. Home deliveries are bound to be un-hygienic, unsupervised and when intervention is required it usually late [2,3]. Despite skilled delivery is one of the most tracked Millennium Development Goals (MDG) indicators, the proportion of births attended by skilled health personnel in Ethiopia and in the region at present is about 6% and 4% respectively [2]. Home deliveries have been associated with adverse infant and maternal outcomes [4-10]. The highest number of maternal deaths occurs on the first day after delivery highlighting the critical need for good quality care during this period [11-17].

Interestingly, a large proportion of these maternal deaths could be prevented through timely and appropriate interventions. The presence of skilled delivery service utilization at each birth can significantly reduce the maternal mortality and morbidity [10,14].

Many studies revealed the presence of positive association between utilization of maternal health care and residence; those living in urban and closest to health facilities tend to utilize skilled delivery services than rural dwellers [18-21]. Maternal education is also considered as most important factor in determining women’s delivery care seeking behavior [22-24]. However, education of mothers may not maintain its effects across all levels of education and social settings [23,25-28].

Knowledge is also an important factor that affects attitude, intension and behavior. Knowledge relates to behavior, and behavior produces change towards service utilization [29-34]. The more knowledge they have about dangerous signs of pregnancy and delivery the more they go for antenatal and delivery services [30]. Studies conducted to assess the relationship between knowledge and skilled delivery service utilization consistently showed that, knowledge is strong predictor of maternity service utilization; those having good knowledge about danger signs of pregnancy and delivery are more likely using skilled delivery services. It is expected that a better informed individual is better placed to make reasonable decisions [34,35]. A strong positive association that has been also shown to exist between quality of care obtained during pregnancy and the use of skilled delivery care [16,18,20,22,23].

In many studies done in Africa, most mothers express their wish to deliver in a health unit [27,31]; in reality, the majority of them end up either not being attended or attended by non trained people (the majority of whom are family members) during delivery [31,33]. Although most pregnancy and delivery related complications cannot be predicted, high quality antenatal care (ANC) and receiving counseling on birth preparedness during antenatal care appeared to strongly influence women’s use of skilled care during delivery [30-32].

In Ethiopia, the utilization health facilities for delivery service still stagnates at lower level despite a rapid expansion of the health sector throughout the country. It believed that there are different factors that operate at different level determining the utilization of delivery service. This study was conducted with the aim of identifying the factors related to low utilization of delivery services.

## Methods

A case control study was conducted in all public health facilities in Bahr Dar Special zone in Ethiopia. The study area comprises one referral hospital, ten rural and urban health centers and their outreach sites. The population of the study area was 240,380 and the annual estimated deliveries were 12,200 deliveries of these 16% took place in health facilities.

The referral hospital in the zone provides comprehensive emergency obstetric care while the health centers provide basic emergency obstetric care. Health extension workers provide clean and safe delivery and referrals at health post. In the zone, immunization service utilization (80%) is higher than delivery service [2,24].

The study participants were all mothers who came for post-natal care or those who brought their children for immunization service. Mothers who gave birth at home for their last pregnancy were considered as case while mothers who delivered at health facility were categorized in the control group.

A two proportion formula *(stat calc* EPI info 3.5.1*)* was used to estimate the sample size required of the study. The following assumptions were made to estimate sample size required for the study. A 95% confidence level and 80% power were used. ANC coverage was used as exposure variable. The calculated sample size to detect an odds ratio of 2.0 and case: control ratio of 1:2. The final estimated sample size was 119 for cases and 238 for controls.

The average number of mothers coming for post natal care and immunization service was estimated based on previous reports from the facilities. The estimated sample was allocated proportional to the case load of each facility.

Mothers who delivered in four months period preceding the data collection were asked about their place of delivery. They were grouped as cases or controls based on their place of delivery. Both cases and controls were selected using systematic sampling technique, every third case and control were interviewed until the allotted sample is reached at each health facility.

Face to face interview using a pre-tested and standardized questionnaire was conducted to collect the data. Questions extracted from DHS & other literatures served to prepare the instrument [2]. The instrument was pretested on 35 mothers who were attending post natal care service in health centers. Findings from the pretest were used to modify the instrument.

Place of delivery was the main dependent variable while characteristics that determine the place of delivery service namely age, number of children, education, knowledge, attitude, residence and quality of care were the independent variables.

Skilled delivery was defined as a delivery that has taken place at health center or a hospital. Home deliveries are considered as non skilled delivery in this study, according the prevailing practice in the country.

Data collectors were nurses and health officers who were selected based on their experience in research work. Supervisors were public health graduates. Training was given for two days. The training focused on understanding the research question, ethical conduct, and quality of data collection. Supervision was made by two supervisors and the principal investigator.

The data was entered into EPI info version 3.5.1. Once the entry was completed it was exported into SPSS version 17. STATA 11 was also used. The data was summarized using tables. Composite scales were constructed for knowledge, attitudes and practice variables that used different items for measurement. Knowledge was assessed using 18 items on danger signs of pregnancy and delivery. Correct responses were given 2 points and 0 point was given for incorrect answers. The final scores were computed to give a composite scale with categories (Good, fair or bad) based on following cut off points (“>75 “good, “≥ 50-74% = fair”, and “< 50% “bad”).

Attitude of mothers was measured using 10 attitude questions. (Example: Pregnant women can choose their place of delivery by themselves, Husbands promote their wives to attend skilled delivery care, Attending skilled delivery service is safe and satisfactory). The response of each mother was given. Finally the response was recoded into “1” if the mother has agreed for the question and “0” otherwise. The total score was dichotomized into favorable and unfavorable attitude.

Practice of mothers was also measured based on prompts to see whether they had adequate maternal health practices. That includes attendance of ANC and frequency of ANC attendance. Accordingly, a score of “1” was given for mothers who fulfill the notion of good practice and “0” point for those who don’t fulfill those criteria. And finally it was dichotomized into good and unsatisfactory practice.

Logistic regression was employed to analyze the data. Crude and adjusted Odds ratios were computed for each explanatory variable to determine the strength of association and to control the effect of confounders. Variables with significant association with the dependant variable were entered into the final model. The model was tested with goodness of fit test yielding LR (chi square calculated = 120 and P-Value = 0.000).

Ethical approval was secured from institutional review board of the University of Gondar. Informed verbal consent was obtained from all participants.

## Results

Among a total of 356 participants sampled, 108 cases and 216 controls accepted the invitation to participate. As shown in Table 1 there was significant difference between cases and controls in their age (χ^2cal^ =10.38,df = 4 and p = 0.034), residence (χ^2cal^ =95.63,df = 1 and p = 0.000), level of education of mothers (χ^2cal^ =89.82,df = 4) and p = 0.000), income(χ^2cal^ =83.01,df = 4 p = 0.000) level of education (χ^2cal^ = 95.4,df = 4 and p = 0.000).

**Table 1:** Socio-demographic and obstetric characteristics of participant mothers aged 15-49, Bahir Dar, 2010

***Variables***	***Cases (n = 108)***	***Controls (n = 216)***	***χ***^***2***^***square,df and p- value***
**Age of the mother**			
15-19	7 (6.5)	14 (6.5)	χ^λαχ2^83.01=
20-24	31 (28.7)	76 (35.2)	df = 4
25-29	34 (31.5)	88 (40.7)	P-value = 0.034
30-34	25 (23.1)	26 (12.0)	
35-39	11 (10.2)	12 (5.5)	
**Maternal education**			
Illiterate	83 (76.9)	54 (25.0)	χ^λαχ2^28.98=
Read & write	8 (7.4)	11 (5.1)	df = 4
Primary	12 (11.1)	77 (35.6)	P-value = 0.0000
Secondary and above	5 (5.0)	74 (34.1)	
**Husband Education**			χ^λαχ2^74.69=
Illiterate	63 (58.3)	29 (13.4)	df = 4
Read & write	17 (15.7)	14 (6.5)	P-value = 0.0000
Primary	18 (16.7)	62 (28.7)	
Secondary and above	10 (9.3)	111 (51.4)	
**Residence**			χ^λαχ2^36.59=
Urban	49 (45.4)	202 (93.5)	df = 1
Rural	59 (54.6)	14 (6.5)	P-value = 0.0000
Average monthly income* n = 248	**n = 55**	**n = 193**	
<1000.00ETB	44 (80)	89 (46.1)	χ^λαχ2^83.01=
1000-1999.00ETB	9 (16.4)	60 (31.1)	df = 4
> = 2000.00ETB	2 (3.6)	44 (22.8)	P-value = 0.000

The following variables were found to be predicators of place of delivery. The likelihood of delivering at home was greater among mothers with inadequate knowledge of pregnancy related problems (AOR = 62, 95% CI: 3, 128.1), those who started attending ANC after 24 weeks of gestation (AOR 8.7, 95% CI: 2.2, 33.3), mothers having no formal education (AOR 4.2 95% CI 1.63, 11.27) and rural residents (AOR = 3.6, 95%CI: 1.4, 9.0) (See Tables 2 and 3).

**Table 2:** Obstetrics characteristics, Knowledge, Attitude and practice of participant mothers in Bahir Dar special Zone, 2010

***Variables***	***Cases(n = 108)***	***Controls(n = 216)***	**Crude OR (95% CI)**
	**No(%)**	**No(%)**	
Para			
1-3	93 (86.1)	179 (82.8)	1.3(0.7, 2.4)
≥4	15 (13.9)	37 (17.2)	1
Abortion			
Yes	15 (13.9)	18 (8.3)	1.8(0.9, 3.7)
No	93 (86.1)	198 (91.7)	1
Still birth			
1-2	3 (2.8)	7 (3.2)	1
None	105 (97.2)	209 (96.8)	0.8(0.2, 3.4)
No of Surviving children			
1-4	103 (95.3)	203 (93.9)	1.3(0.4, 3.8)
5-8	5 (4.7)	13 (6.1)	1
Knowledge about danger signs of pregnancy			
No	39 (36.1)	33 (15.3)	**3.1(1.8, 5.4)**
Yes	69 (63.9)	183 (84.7)	1
Knowledge about danger signs of Labor			
No	23 (21.3)	12 (5.6)	**4.6(2.2, 9.6)**
Yes	85(78.7)	204 (94.4)	1
General knowledge about obstetric complications			
Bad	22 (20.4)	2 (1)	**27.9(6.4, 122)**
Fair	18 (16.7)	41 (19)	1.1 (0.6, 2.0)
Good	68 (62.9)	173 (80)	1
Over all attitude			
Unfavorable	88 (81.5)	108 (50)	**4.4(2.5, 7.6)**
Favorable	20 (18.5)	108 (50)	1
Last pregnancy planned			
No	25 (23.1)	38 (17.6)	1.4(0.8, 2.5)
Yes	83 (76.9)	178 (82.4)	1
Got ANC for last pregnancy			
No	51 (47.2)	11 (5.1)	**16.6 (8.1, 34)**
Yes	57 (52.8)	205 (94.9)	1
Got any advice on where to deliver			
No	62 (57.4)	52 (24.1)	**4.2 (2.6, 6.9)**
Yes	46 (42.6)	164 (75.9)	1
Number of visits			
1-2	17 (29.8)	26 (12.7)	**2.9 (1.4, 5.8)**
3 or more	40 (70.2)	179 (87.3)	1
Counseling			
No	27 (47.4)	43 (21.0)	**3.4 (1.8, 6.2)**
Yes	30 (52.6)	162 (79.0)	1
Satisfied with ANC care			
No	11 (19.3)	12 (5.9)	**4.5 (1.8, 10.9)**
Neutral	7 (12.3)	2 (1.0)	0.2 (0.04,1.54)
Yes	39 (68.4)	191 (93.1)	1
Over all ANC quality			
Inadequate	49 (86.0)	160 (78.0)	1.7 (0.7,3.9)
Adequate	8 (14.0)	45 (22.0)	1
Who decided place of last delivery			
Husband	20	49	1.5 (0.8, 2.8)
Others **†**	17	42	1.5 (0.8, 2.9)
Together	13	32	1.5 (0.7, 3.2)
My self	58	93	1

† Include mother or grandmother or mother in-law.

**Table 3:** Predictors of home delivery

***Variables***	***Number of***		**Odds ratio**
	**Cases = 108**	**Controls = 216**	**Adjusted OR (95%CI)**
**Maternal Education**			
No formal education	91	65	4.27 (1.63, 11.27)*
Formal education.	17	151	1
**Address**			
Rural	59	14	**3.6 (1.4, 9.0)** *
Urban	49	202	1
**General knowledge about obstetric complications**			
Poor	22	2	**62 (3, 128.4)** *
Fair	18	41	0.7 (0.2,1.7)
Good	68	173	1
**Time of first ANC visit**			
< 12 weeks	8	58	1
12-24 weeks	35	140	1.6 (0.7, 4.0)
**After 24 weeks**	14	7	8.7 (2.2, 33.3)*

*Adjusted for Maternal education, Husband education, Residence, Knowledge on danger sign of pregnancy, Attitude, and Time of ANC visit.

Bahir Dar, Special Zone 2010.

## Discussion

The study identified very important predictors that are related to geographic access such as living in rural area; social factors such as being illiterate; lack of access to information such as having poor knowledge about obstetric complications and delay in starting first ANC visit.

The recall period in the present study was maintained at 4 months or less than 4 months, that should be counted as strength of the study.

Among the social factors, education of mothers appeared to be the most important predictor in determining the utilization of institutional delivery care after controlling other variables. Many previous studies conducted in developing countries have found education of mothers to be among the most important determinants of skilled delivery care utilization [20,21]. There are a number of explanations that speculate as to why education is a key determinant of skilled care demand. For example education is likely to enhance female autonomy so that mothers develop greater confidence and capabilities to make decision regarding their own health, as well as their children. It is also more likely that educated women demand higher quality service and pay more attention to their health in order to insure better health for themselves. Moreover, educated women are more likely to be aware of difficulties during pregnancy and as a result, they are more likely to use maternal health care services [20].

Another predictor that has also shown an important influence on maternal health care utilization was place of residence. Mothers living in rural area were less likely to use institutional delivery services than urban mothers. This finding is consistent with the previous studies done in Ethiopia, Kenya and Uganda [8,26,27], which suggested that place of residence has a statistical significance variable on the use of skilled care. It is consistent with the fact that rural mothers have limited financial and transport access to receive institutional delivery service [28]. Similarly according to the data from DHS report (2005) [2], large disparities exist in many key health indicators in relation to place of residence, signifying that living in a rural area is a barrier to seek modern health care.

Having poor knowledge about pregnancy and delivery for instance was strongly associated with home delivery. Similarly having lack of knowledge about danger signs of pregnancy was very strongly associated with home delivery.

This finding demonstrated the fact that obstetric knowledge is an important factor that affects attitude, intention and behavior of mother. [22,29,30]. Another explanation for this could be knowledge of danger signs of obstetric complication is the first step in the appropriate and time referral for essential obstetric care [22]. Moreover the main reasons leading to poor use of skilled care services include the personal belief, knowledge, attitude and life style of pregnant mother [31].

These findings are consistent with those of previous studies in Ethiopia, Ghana, Kenya and Vietnam [8,21,27,28] respectively. The more knowledge they have about the importance of skilled obstetric care the more likely they have a positive attitude towards skilled obstetric care utilization [28,32].

In this study, level of antenatal attendance was high (80.9%) and this figure is higher than the national or regional estimate [8,25,34,35], the reason for this disparity may be due the high number of mothers from urban residence. those mothers who delayed presentation for ANC until the end of second trimester were more likely to attend home delivery than those came earlier. This can be best explained by the fact that ANC is more effective when received earlier in the pregnancy. This finding is consistent with previous studies [20,33,36].

In this study women who did not receive counseling where to deliver were more likely to deliver at home in the bivariate analysis. This result wasn’t retained in the multivariate analysis. Failing to counsel women about preferred place of delivery during antenatal care can be considered as a missed opportunity to improve the rate of skilled deliveries.

With regard to other correlates of skilled delivery care utilization, our finding did not reveal any supporting evidence that age, monthly income, number of surviving children and history of abortion to show a statistically significant difference between cases and controls. These findings could be related to difference in research methodology, sample size and the difference in other social and demographic factors that might not be accounted in this study.

Case control studies are not able to establish temporal relationships. So it is difficult to establish causal relationship in this study.

A non-response rate of 6% also affects estimate of a parameter and power of a test. Selection bias may be inevitable since cases and control were selected among mothers who only visit postnatal care or immunization for their children, this may affect generalizability and internal validity. Those who have limited access for all kinds of service may not be included in the study. This may result in differential misclassification obscuring some potential differences.

Those who delivered at heath facility might have been forced to visit care because of presence of serious clinical conditions during pregnancy and childbirth.

Tools used to measure knowledge and attitude were not standardized and tested for their reliability which would affect its comparability.

Inclusion of other variables like perceptions of the existing quality of delivery service, direct and indirect cost of health services and ability of the participants to pay for the service could have provided a comprehensive picture.

## Conclusion

The predominant factors associated with not utilizing skilled delivery services in the study area are lack of knowledge about obstetrics care, delay in starting Antenatal Care (ANC) visit, and low level of education. Place of residence was also an important predicator of place of delivery.

## Recommendations

Audience specific behavioral change communication should be designed to improve the demand for delivery services. Health professionals should take the opportunity to encourage mothers attend delivery services during ANC follow up. Improvements should be made in social conditions including education and major social mobilization endeavors. To have a complete picture of the situation, it is recommended to assess the quality of delivery service, perception of the potential users on the quality of care and other cultural factors.

## Competing interests

The authors declare that they have no competing interests.

## Authors’ contributions

F A has participated in the design, coordination of the study, data collection, statistical analysis and development of the draft manuscript. Y B has participated in the design, coordination of the study, statistical analysis and helped to draft the manuscript. B G has participated in the design, coordination of the study, statistical analysis and helped to draft the manuscript. All authors read and approved the final manuscript.
